# Fostering Vicarious Resilience for Perinatal Palliative Care Professionals

**DOI:** 10.3389/fped.2020.572933

**Published:** 2020-10-08

**Authors:** Kathryn R. Grauerholz, Michaelene Fredenburg, Premala Tara Jones, Kristy N. Jenkins

**Affiliations:** ^1^Life Perspectives, San Diego, CA, United States; ^2^Counseling and Testing Center, University of Akron, Akron, OH, United States

**Keywords:** perinatal, palliative, compassion, burnout, ethical, self-care, grief, resiliency

## Abstract

**Background:** The demands on healthcare professionals caring for families grappling with a life-limiting condition in an unborn or newly born child can be overwhelming. Clinicians working in emergency/trauma, hospice, and pediatric settings are already at high risk for burnout and compassion fatigue, which can leave healthcare institutions increasingly vulnerable to poor retention, absenteeism, and waning quality of care. The provision of exemplary palliative care requires a cohesive interdisciplinary team of seasoned professionals resilient to daily challenges. In September 2019, the American College of Gynecology, in a committee opinion, published standard of care guidelines for perinatal palliative care. This has created an impetus for exceptional caregiving and a greater demand for both physician and interdisciplinary healthcare provider education, training, and ongoing support that promotes truly beneficent care for pregnant patients confronted with life-limiting fetal conditions.

**Methods:** A scoping review of the research literature was conducted in order to distinguish the barriers and facilitators of professional resiliency in perinatal palliative care. PubMed, Medline, CINAHL, and EBSCO Psychology & Behavioral Sciences Collections were systematically reviewed. Because of the paucity of studies specific to perinatal palliative care, several interviews of nurses and physicians in that field were conducted and analyzed for content distinctly pertaining to personal practices or workplace factors that support or hinder professional resiliency.

**Results:** The research indicated that medical professionals often cite a lack of knowledge, inexperience using effective communication skills related to perinatal palliative care and bereavement, challenges with interdisciplinary collaboration, misconceptions about the role and function of palliative care in the perinatal or neonatal settings, moral distress, and workload challenges as encumbrances to professional satisfaction. Strategic implementation of facility-wide bereavement care training, effective communication modalities, and evidenced-based practical applications are critical components for a thriving perinatal palliative care team. Authentic formal and informal debriefing, peer mentoring, adequate caseloads, robust provider self-care practices, exceptional relational efficacy, and cultural and spiritual humility can foster personal growth and even vicarious resilience for perinatal palliative care professionals.

**Conclusions:** Support should be strategic and multifaceted. The onus to implement salient measures to cultivate resilience in the perinatal palliative caregiver should not be only upon the individuals themselves but also upon prevailing regulatory governing bodies and healthcare institutions.

## Introduction

Perinatal palliative care professionals have an integral role in the decision making and care delivery for many families encountering uncertain perinatal outcomes, some of which may have a reverberating and lasting impact. Palliative care champion Byock (1), in his 1997 book *Dying Well*, described how the time preceding a death can be a time of personal and spiritual growth for many. Researchers Côté-Arsenault et al. [(2), p. 12] said that “contrary to common societal reaction where focus is on what is wrong with the baby, [the study participants] focused on everything positive about their babies.” The parents also said that their participation in the research study was a positive experience and added meaning to their children's short lives (2). Nurses in a study that explored their reactions to perinatal death revealed that when adequately supported, “growth and transformation” emerged from “the anguish and grief” (3). This article will systematically explore ways that professional caregivers can glean a fecundate resilience and vicarious personal growth sojourning with their patients who are enduring a life-limiting fetal diagnosis.

The loss of a child in infancy can be a profoundly traumatic experience. Psychologists Jaffe and Diamond (4) described in their 2011 book, *Reproductive Trauma: Psychotherapy with Infertility and Pregnancy Loss Clients*, the hopes and dreams that individuals envision for their future family long before conception. They also portrayed the trauma that can occur when those hopes and dreams go awry. The psychological ramifications when one's fundamental beliefs about themselves and their offspring are disrupted by a life-limiting antenatal diagnosis can be devastating. Lang et al. (5) demonstrated in their research that the death and dying of unborn or newly born children, though culturally disenfranchised, is as emotionally painful as the loss of an older child or adult family member. The researchers also concluded that because of the lack of social support, intrinsic to disenfranchised losses, parents' emotional reactions to perinatal losses tend to be particularly traumatic compared to more traditional types of losses (5). Doka [(6), p. 5] described disenfranchised grief as an experience that “is not openly acknowledged, socially validated, or publicly observed.” The birth and/or untimely death of an infant and the associated grief reaction is outside of typical grieving norms, and parents can find themselves on an unworn path of bereavement.

The emotional impact on families confronted with a life-limiting fetal illness is often complex. At the time of diagnosis, parents may experience a variety of emotions including fear, shock, anger, anxiety, sadness, guilt, and even jealousy of other parents with healthy babies (7). The risk for severe psychological distress including depression, anxiety, and obsessive-compulsive behaviors was shown to be as much as five times higher in parents with a very low birth weight infant in comparison to parents with normal birth weight babies (8). In addition, some families have to cope with financial strain, time away from other family members, long commutes, and travel expenses (8). Furthermore, a couple planning a delivery or termination of a fetus with a life limiting condition is likely to encounter disagreements about their decision from healthcare professionals, family members, and friends. In the absence of multidisciplinary planning and support, the antenatal and postpartum bereavement has the potential to be fraught with ambivalence, shame, secrecy, and isolation. Those involved in the care provision may also find that, in the absence of personal resiliency practices, the persistent emotional strain is unsustainable (9, 10).

Palliative care teams, comprised of multidisciplinary professionals serving inpatient and home environments, are a relatively new concept in medical practice, particularly in pediatrics, and perinatal care. The team usually consists of two or more interdisciplinary professionals with complementary skills who collaborate and move toward common focused goals in the delivery of care (11). Team members often depend on and hold each other accountable for different complex and mutable aspects of palliative care. Perinatal teams are structured like adult and pediatric palliative care teams, which usually include medical care providers, social workers, chaplains, respiratory therapists, and lactation consultants. It is recommended that nurses and physicians in these teams be trained in obstetrics and neonatology (12). Because of improved technology and the earlier diagnoses of genetic anomalies and lethal malformations over the past four decades, expectant couples are now being counseled about the health of their unborn child earlier in the pregnancy. Since the onset of antenatal prognostication, the option of pregnancy termination was initially purported as the only measure to avoid suffering, and comfort-focused alternatives were not developed nor presented as a modality of care (13). Now, couples are being offered a broader spectrum of care that includes palliative and hospice provisions for their unborn (14). Thus, frequent acquiescence of the palliative professional's approach to varied patient care situations is essential, and enduring occupational resiliency is indispensable.

## Methods

**Figure 1:**
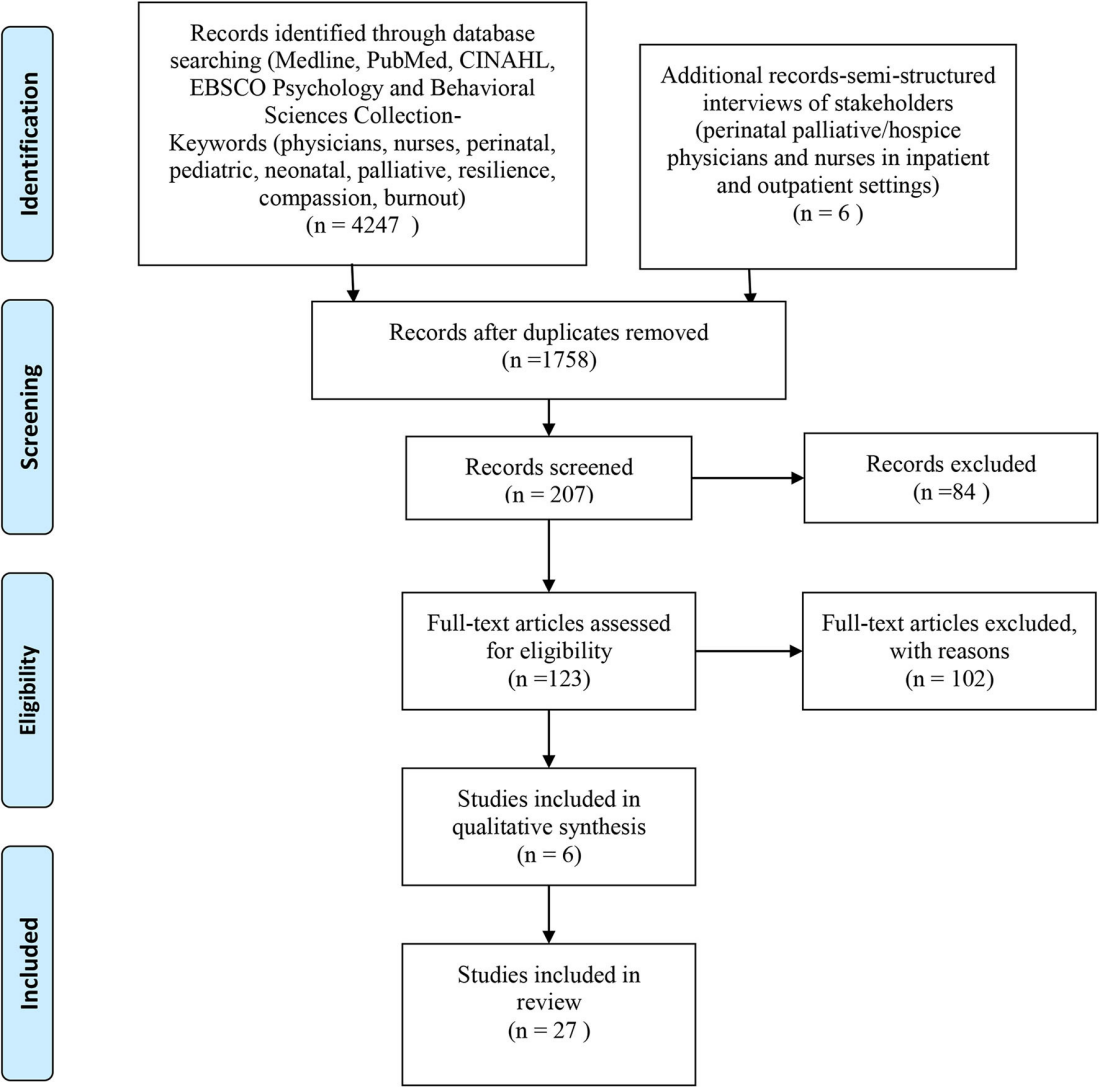
Flow diagram of article section process.

A scoping review according to the methods described by (15) was used to explore and amplify the key factors necessary for promoting professional stability in the perinatal palliative setting (Figure 1). The scope of practice for perinatal palliative care is still evolving, but for the purposes of this study the field of reference was that outlined by the American College of Gynecology (14). There is a body of research that explores various aspects of self-care and supportive measures to prevent compassion fatigue and burnout in healthcare providers in pediatric and palliative settings, but there is a paucity of studies specific to perinatal palliative care. The search included systematic reviews, qualitative case studies, and quantitative survey studies that had been published in the past 11 years (January 2009–January 2020) (Figure 1). Searches were made in PubMed, Medline, CINAHL, and an EBSCO Psychology & Behavioral Sciences Collection. Search words included *perinatal, neonatal*, and/or *palliative* combined with the words *burnout, compassion fatigue*, or *resilience*. Relevant studies were identified from screening summary and abstracts. Full articles were analyzed for components associated with professional caregiver burnout, compassion fatigue, resilience, and satisfaction (Table 1). In addition, semi-structured interviews were conducted (Table 2). The participants included four registered nurses and two physicians involved in various perinatal palliative and hospice care programs in the following US cities: San Diego, CA; New York, NY; Denver, CO; and Flagstaff, AZ. The interview questions were emailed to the perinatal palliative professionals who had agreed to participate. They either composed and returned written responses or answered the questions in a phone call conducted by the primary author, which were then transcribed. The responses were aggregated and then the content was analyzed for facilitators and barriers to professional resilience by two coders who have graduate level education in nursing or psychology (Table 3). There was no specific funding utilized for the conduction of this study.

**Table 1:** Study descriptions and details.

**Author(s)**	**Year of publication**	**Aim of Study**	**Methodology**	**Findings summary**
Back et al.	2016	Examine the approaches to building resilience in palliative care physicians—clinician resilience skills & workplace factors	Literature review and stakeholder interviews	Introduced a structural model of individual skills and system construction that support professional resilience
Cortezzo et al.	2019	Study of birth planning for life-limiting fetal diagnosis from both the patient and parents' perspectives	Mixed methods, quantitative-descriptive & univariate analysis; qualitative-thematic analysis	Proposed eliciting parental preferences for greater participation and decision making. Physician comfort levels were adequate, but found that time limitations made communication aspects with birth planning was most unfeasible aspect
Cortezzo et al.	2015	Explore the practices and perceptions of end-of-life care in NICU—examined patient and provider experiences	Survey study- explorative, description cross-sectional evaluation	Found debriefing/closure conferences, bereavement support, and education provision was variable and suboptimal at times. Value of formal palliative care teams rather than individual consultants ascertained.
Forster and Hafiz	2015	Explore the coping strategies and perceptions of support available to professionals encountering pediatric death	Qualitative: multidisciplinary professional interviews	Personal coping strategies, peer support, family support, spiritual beliefs, relational efficacy when caring for the dying child and family all improved coping
Hamric	2014	Illustrate ethical issues and moral distress associated with end-of-life issues that occur in the NICU which impact perinatal palliative care provision.	Case study	Expounded ethical climate building with debriefing, improving communication, multidisciplinary care conferences, and resilience factors
Hutti et al.	2016	Evaluate nurses reactions to caregiving for patient experiencing fetal demise	Recorded group discussions structured by Swanson's Theory of Caring was analyzed	Risks for compassion fatigue were revealed. Coping strategies and interventions based on latest research was presented which included debriefing, mentoring, more perinatal bereavement education, and self-care practices were implicated.
Jonas-Simpson et al.	2016	Explore thematic patterns of obstetrical or neonatal nurse's grief experiences to perinatal death	Qualitative study: interpretive phenomenology	Thematic patterns included: Growth and transformation emerging with anguish and grief, collegial supports, education, aspects of reciprocal mentorship, the intrinsic value of compassionate caregiving
Kamal et al.	2016	Examine the prevalence and predicting factors associated with burnout rates in hospital and palliative care clinicians in the U.S.	Quantitative: Survey study and statistical analysis	Higher rates of burnout correlated with emotional exhaustion and depersonalization, working at smaller organizations, extended work hours, age < 50, and working weekends. Activities related to interpersonal relationships and vacation use were associated with less burnout rates.
Klein	2009	Study issues of ethical dilemma and moral distress in pediatric palliative care—infants born with severe life-limiting anomalies	Case studies	Prognostication challenges, cultural, and social factors can cause moral distress in parents and palliative staff. Beliefs and aspects regarding the suffering of the infant can create emotional dilemma. Ethics and palliative care collaboration can be supportive to staff and caregivers.
Kilcullen and Ireland	2017	Present facilitators and barriers to palliative care provision in an Australian NICU	Qualitative: Thematic analysis of semi-structured interviews	Facilitators of the provision of palliative care included: cultural awareness, experienced and proficient mentors and supportive staff, clinical skills and knowledge, personal knowledge of one's own values, morals, & beliefs, emotional & communication skills, and knowledge of bereavement practices. Barriers to the provision of palliative care included: lack of education/inservices, workspace environment, parents' inaccessibility to infant, nurses' own grief, and included input into development of policies and procedures.
McCloskey and Taggart	2010	Probe the occupational stressors of pediatric palliative care nurses	Qualitative focus group discussions: thematic content analysis	Workload demands included: emotional, ethical, constraints to care delivery, lack of resources, documentation, community misconceptions, maintaining relationships with patient & family, and cultivating autonomy within the profession
Mehta et al.	2016	Study aspects of palliative care teams- pilot study of model for team resilience	Quantitative- Resiliency model testing with physicians	Team building measured a feasible reduction in perceived stress, and improved the perspective taking of the others in the team
Mills et al.	2017	Evaluate perceptions, education, and practices related to self-care in palliative nurses and medical professionals in Australia	Survey studies	Self-care was regarded as important by most. Many had not practiced self-care and also denied having any education on self-care. A large portion of those reported that self-care plans would be considered if training was provided.
Mills et al.	2018	Explore the meaning and practice of self-care in palliative nurses and physicians	Qualitative content analysis of semi-structured interviews	A practice of self-care contributed to a better capacity to care for others. Self-care practices in both occupational and personal settings is necessary.
				Facilitators of self-care practices included supportive environments, self-assessment, prioritization, and an ongoing planning process. Barriers included overwhelming workload & excessive busyness, stigma against self-care at the workplace, low self-worth or self-criticism, and lack of planning.
Perez et al.	2015	Examine the barriers and facilitators of resiliency in palliative care clinicians	Qualitative: content analysis of semi-structured interviews	Identified stressors, coping strategies, and training needs. Stressors included limited resources & cost-cutting, conflicting interdisciplinary expectations, increased caseloads, intensity of the cases, boundary setting, personal & professional limitations, competing demands from administrative & documentation activities, patients, & staff. Coping strategies included personal self-care, emotional & physical distancing from work off the clock, and social & emotional supports. Training needs included mind-body skills, stress education, cognitive stress management skills, and brief coping strategies to implement in real time.
Profit et al.	2014	Explore aspects of burnout and resilience in NICU	Cross-sectional surveys	NICUs with higher levels of burnout were correlated with a reduced teamwork climate, safety climate, job satisfaction, perceptions of management, and working conditions. A resilient culture was show to enhance safety and quality of care.
Sanso et al.	2015	Determine the relationship between self-care & awareness and development of burnout, compassion fatigue, and coping with death in palliative care professionals	Quantitative- survey study	Self-care and self-awareness improved coping with death and compassion satisfaction, less burnout and compassion fatigue. The data collection after the spiritual training exercises did not have those desired effects.
Dev et al.	2018	Evaluate the relationship between barriers to compassion and burnout in nurses.	Quantitative study: survey	Barriers to compassion, contributors to burnout included workload, clinical barriers, practice of self-compassion, encounters with patient and families
Weitraub et al.	2016	Contrast physician burnout, compassion fatigue, and compassion satisfaction	Quantitative study: linear regression analysis	Overlapping of burnout and compassion fatigue found. Impacts emotional well-being and professional performance of neonatologists. Self-identifying as being Hispanic debriefing and utilization of pediatric palliative care services resulted in compassion satisfaction.
Wright et al.	2011	Examine the facilitators and barriers to the provision of end-of-life care in the NICU settings from the perspectives of neonatal nurses.	Quantitative: Questionnaire survey study	Five barriers were identified as: inability to express values and beliefs regarding palliative care, environmental constraints, engagement in technology, demands from parents, and lack of education. Eight facilitators identified as: staff support, involvement of parents in decision making, support from medical team when palliative care was implemented, parents informed of options, staffing, time allotment with dying baby, policies/guidelines that supported palliative care, and available counseling.
Zwack and Schweitzer	2013	Analyze stakeholder interviews regarding aspects of burnout and resilience	Qualitative study: Concept analysis	Both attitudes and strategies (social, mental, and physical) assisted physicians' success to augment decision making, coping, and promoting resilience in others.

**Table 2:** Interview questions based on initial research review.

Please avoid including any patient identification information that would violate HIPAA regulations.
1. Tell me about your experience as a healthcare professional involved in caring for pregnant patients confronted with life-limiting fetal conditions?(Certification, length of time, positions)
2. Are there any particularly memorable experiences when you felt the care received by the patient and the child was at its best?
3. Can you recount an experience when you felt that the care and/or prognostication process were less than ideal for the situation?
4. How often does it seem like there is one or more members on the palliative/hospice care team that are in disagreement with the plan of care? (An estimate of average amount of time)
5. Have there been times when you disagreed with the plan of care and felt morally conflicted by the care given? Do these situations occur more often with particular diagnoses rather than others?
6. What types of interdisciplinary issues seem to cause the most conflict in creating and carrying out a plan of care?
7. Does it ever seem like residue of past distress over patient care experiences effects the quality of care that you provide now?
8. Does it seem difficult to keep a cohesive and effective palliative care team together? What do you think are the barriers or challenges?
9. In what way have you changed as a provider than you were when you began providing perinatal palliative care/hospice?
10. Is there someone who was a mentor to you when beginning this type of work? Now?
11. What did the mentor do that was helpful to your professional development?
12. Do you have particular self-care practices that are helpful in managing work related tension or distress? How long have you been practicing these?
13. Does your employer provide any supports to well-being at work or for off-work hours?
14. Do you have any ideas about methods of self-care that could be provided in the work place or by your employer that would be beneficial to employee well-being?

**Table 3:** Pertinent, categorized quotations from palliative professionals' interviews.

**Palliative Care Collaboration** “The family and staff became well acquainted with the [palliative] goals of care and the relationship with the palliative established prior to the delivery.” “A patient with a complex relationship with [her] partner was recently handled well with multi-disciplinary support we had available for her.” “The social worker continued contact and visits with family, providing psychosocial support, birth plan guidance and memory making tools.” “The baby was stillborn, and parents' needs were met, however I felt they would have had more support if they had let the team be more involved.” “We have a great deal of respect for each other. We have extreme trust in each other. We all are in agreement about the mission and vision of our program.” “Most professionals in other disciplines (speech pathology, lactation consultants, chaplains, and child-life therapist) admire the work of our program, moreover there are some who are very comfortable with palliative care and help us a lot facilitating comfort for babies and support for parents. I believe that no professional in our institution has been in conflict with our program. Most obstetricians have come along to supporting us after seeing the benefits and appreciation from the patients of what we have done for them.” **Moral, Ethical Issues, and Misconceptions** “The mother was a nurse and asked for oxygen and pain meds to prevent her baby from suffering. Although the healthcare team knew this to be unnecessary nor beneficial, we accommodated the mother's request to empower her during this time. By doing so, she was able to have the motherly control to help her during the grieving process.” “More disagreements occur with other specialties like maternal-fetal medicine physicians or when staff members are not understanding role of perinatal palliative care.” “[Conflict] often occurs because medical professionals feel compelled to suggest abortion whenever there is an anomaly.” “Sometimes the parents choose not to feed the baby, even if feeding might be tolerated.” “Sometimes other professionals have questioned our plan of care because they have a different mindset and are not familiar with a palliative care approach. However, when they see the results, the comfort of the baby and parental satisfaction, they come along and they want to learn more about it.” “Mostly the conflicts are ethical. Many team members are utilitarian in their ethical approaches, not out of malice but due to the prevailing utilitarian ethic in medicine.” “There was one situation where the baby was born severely malformed and the parents had the school age children take pictures with baby who did not have severe brain malformation covered. My concern was the internal difficulties the children may have had with viewing the malformation, although externally they presented as accepting.” “In many institutions, babies with life-limiting conditions are not fed at all, we prefer to offer food for comfort, unless the baby is in end of life stage. The experience of feeding their baby is very important for parents, we had babies who lived for only a couple of days successfully breast-fed. We also strongly believe that is comfortable to end life with a ‘full stomach'.” “Sometimes bedside caregivers are morally conflicted and concerned about the futility of care and suffering.” **Professional Strength** “I think each patient's experience is different. Their needs are catered differently. Providing that compassion for the families actually allows me to use my vocation as a nurse to my utmost ability. I think that's when my quality of care is the strongest.” “Occasional vacancies are difficult in the neonatal intensive care unit and palliative programs.” “I have become more open minded and attentive. I look at each single patient and look to them to tell me through objective signs and cues as to what to do for them. It is a tremendous gift to learn from my little patients and from their parents.” **Educational Strategies** “Field staff are offered presentations and webinars including those on compassion fatigue and burnout.” “Bedside staff [report] not feeling comfortable with nature of palliative care. [It is because of] a lack of training and experience. [They are] used to curative goals.” “The perinatal palliative program was difficult when it was located on the postpartum unit, because the nurses had a hard time transitioning to a palliative approach.” “Babies die differently than other children and the outpatient hospice nurses not always educated to the differences. For instance a baby with trisomy 18 will have a different breathing patterns than typical counterparts.” “My previous experience taking care of hospice patients or elderly as they passed was quite different than taking care of families that lost their babies. The grieving seems to have different, complex elements to it.” **Formal and Informal Debriefing** “Our team works very well together and we have discussions, planning, and debriefing as a team. We are friends, a tight, knit group, and are not afraid to express concerns to each other.” “Debriefing is a huge benefit to me. I find that debriefing immediately after a case before going home helps me to relieve that pressure in my heart and mind. It helps me learn from mistakes and set goals for myself for improvement. I also like to call a friend that understands my line of work to debrief with.” “Personally, I think a standardized debriefing practice after unexpected events or difficult situations can promote/be connected to self-care. It is possible that until a situation is debriefed and talked through do healthcare workers really realize how they are feeling about a situation.” “The hospital provides more formal team debriefing on caseloads and challenging cases- increasing and improving these processes.” “In addition, debriefing is essential. It can be a formal debrief or decompressing with a coworker about what happened, how I feel about it, etc. The process of ‘talking things through' not only helps me digest the situation, learn from it and grown as a nurse.” **Care Delivery Models** “In the world of [maternity care], we are able to provide the time and compassion to help families during these difficult times. Fortunately, fetal demise or palliative care is staffed as a one-to-one care.” “The family expressed their gratefulness afterward that they were able to spend as much time with their baby prior to her death at home, and that her last days were not spent in the hospital. Parents were especially grateful for having avoided the surgery because clearly her death was associated with brain stem anomalies.” **Self-Care Initiatives** “This realization can point to the need of self-care and additional supportive interventions.” “They have Reiki, chair massages, hand massages. However, these work best for the people in the office.” “Yes, they provide mental health and debriefing sessions upon request. Also, our clinical manager is very supportive in terms of allowing time to grieve or leave of absence if needed.” “My previous employer and management team were always very supportive and had definitely cared about the overall well-being of the staff. It helps that nurse managers and directors know what it's like as a floor nurse and the difficult situations surrounding [perinatal care] so they know from experience the importance of support, self-care and promoting overall well-being of their staff.”
“[My employer] offers plenty of opportunities for employees such as arranged lunches for nurses and physicians, even nicely arranged coffee breaks. They also offer different exercise programs, yoga, and counseling services. I haven't participated in any of these, I believe because I have so much help through my team and my friends.” “[Our routine team] meetings have time for bereavement and support. Additionally, we have an employee assistance program.” “[I] exercise, garden, and do a lot of home maintenance. I am also very involved with family, grandchildren that live nearby.” “Mostly, I enjoy the friendship of many people. My friends care for my psychological needs. I also sing in a choir.” “I have been practicing self-care all of my career. However, am much more organized now in active planning of self-care. Spiritual discussions and personal involvement with an excellent small group of very close friends (most of them work in hospice) and family; owning and interacting with dogs; daily walks and stretching exercises; massage and chiropractic care; keeping up with medical and dental routine care and issues (years ago I delayed these until they became urgent). My belief is that one can only do any type of hospice and palliative care with a wide variety, and regular, self-care activities.” **Cultural and Spiritual Humility** “The family was also able to sing praise and worship songs to their lovely daughter and love on her.” “I appreciated having continuity and the experience of being a part of a spiritually uplifting situation observing the parents' joy in the life, albeit short, of their son.” “We also rely on the parents to tell us what they think their child needs. They are more intuitive about their own child's needs.” “Honestly, I pray to Mother Mary a lot during my times of distress. If anyone understands what these patients are going through, it's [Mary]. I pray that she helps and guides me as well.” “[An] anencephalic baby delivered at hospital before I arrived. I baptized the baby, [as the parents were] fearing that a priest or deacon would not arrive in time. We provided comfort to the baby including time on breast.” “I keep in mind that ‘I am not God'. I cannot solve everything. Our time is finite, just a drop in the ocean.” “Taking into account their cultural background also is important. Sometimes, the conflicts occur within the partnership of the parents and their cultural differences.”

## Results

Database searches revealed 4,247 studies. Initial review and removal of duplicates, unavailable full-text article, marginal relevance to perinatal palliative care, and language barriers reduced to the search to 123 articles. Another 102 were removed because they did not report primary research. Therefore, 21 articles were selected. The literature review process and content analysis of the interviews yielded the following factors that contribute to distress in neonatal and palliative care professionals: challenges associated with both multi- and interdisciplinary collaboration, gaps in training, moral and ethical issues, misconceptions about the role and function of palliative care in the acute setting, and unfeasible workloads. Alternatively, components that supported resilience and career satisfaction included more comprehensive and interdisciplinary bereavement care training, formal and informal debriefing, supportive measures to strengthen teams and care delivery models, individualized self-care measures, and a humble approach to care provision that included both cultural and spiritual humility.

## Discussion

### Palliative Care Collaboration

Professionals agree that initiating palliative care and education to the family as soon as possible after the devastating diagnosis procures more salient comfort measures, which, in turn, enhances the significance of the care received (16). Care demonstrated to be effective and relevant can improve caregiver satisfaction and resilience. A registered nurse and clinical coordinator of the Neonatal Comfort Care program at New York's Presbyterian Morgan Stanley Children's hospital, Fran McCarthy, recalled a recent case involving a mother who had just received the initial introduction with the comfort care program and staff when she delivered prematurely. A birth plan and end-of-life options and procedures had not yet been reviewed, nor had there been time to develop a trusting alliance. Consequently, the attending medical resident and nurses were distressed about the despondent mother's demands for continued and prolonged resuscitation measures that were not typically indicated for an infant born in the presented condition. The difficult timing issues resulted in a knowledge gap that, in turn, caused parental and staff distress. In contrast, McCarthy recalled that some of the cases she considered to be most optimal had been those in which the staff and the families had adequate time to prepare and nurture a reciprocal relationship prior to the birth of the child. Karen Christie, Registered Nurse Pediatric/Perinatal Case Manager at The Elizabeth Hospice in San Diego, also recalled that more affirming bereavement experiences occurred for families who had received maximum in-depth training and antepartum palliative care planning. In regard to one memorable encounter, Christie said, “I appreciated having continuity and the experience of being part of a spiritually uplifting situation observing the parents' joy in the life, albeit short, of their son.”

Bereavement care initiated soon after an ominous prenatal diagnosis can address the anticipatory grief that parents often experience and help them cope with a loss that is typically multifaceted (17, 18). Perinatal palliative care teams aim to mitigate the trauma of disenfranchised grief through supportive modalities, ongoing education, and active care for patients and their families throughout the perinatal and bereavement periods (12, 19). In several studies (2, 20–22), Côté-Arsenault demonstrated how professionals providing effective family-centered palliative support can potentially optimize the parents' adjustment to the painful event. Many parents also reported feeling fulfilled and even joyful to have had the short time to parent and love their severely ill children (2, 21).

### Moral, Ethical Issues, and Misconceptions

Palliative, neonatal, and obstetric care teams have had to become increasingly flexible to varying patient values and desires for the trajectory of their pregnancy. The increasing scope in the range of care delivery options has not occurred in the absence of moral distress experienced by those providing the care (23–25). When interviewed about the barriers and challenges to keeping a cohesive and effective palliative care team together, the medical director of the Perinatal/Pediatric Hospice Program at The Elizabeth Hospice and a family practice in San Diego, Dr. George Delgado, said the “challenges include emotional stress, compassion fatigue, ethical dilemmas, and cost-cutting across healthcare.” From his experience most of the conflicts are ethical, extending from contrasting societal philosophical axioms influencing healthcare, which can leave professionals in an ideological betwixt and between in the delivery of care. Ethical dilemmas not only surround the termination of a live fetus but also accompany the initiation and continuation of life-sustaining treatments that can be perceived as a prolongation of suffering and an exercise in futility for an infant bound to die (24, 26, 27). Both options are indicated as palliative options on the spectrum of services for those diagnosed with life-limiting fetal malformations by the American College of Obstetrics and Gynecology (14). Emerging perinatal palliative care programs are involved in the provision of comfort care to the neonate that neither aims to hasten death nor aggressively prolong life.

Additionally, moral distress can occur when professionals experience reluctance advocating the interests of their patients for fear of discord with the parental prerogative or institutional constraints (28, 29). Practitioners delivering direct patient care in acute situations are more vulnerable to moral distress, which can lead to burnout and staff retention issues (30, 31). Strategies for addressing and alleviating moral distress include frequent communication with the family, clarifying patient understanding of diagnosis/prognosis, engaging in ongoing goal setting, discussing the ethical issues, increasing team collaborative efforts, and using a skilled ethics facilitator for high levels of distress (23, 32).

Misconceptions surrounding the goals and spectrum of palliative care are often the most challenging aspects of its provision and collaboration amid the dominant curative culture of medicine. Caregivers may also deduce that their service efforts are marginalized when the modality of palliation is disparaged within the healthcare system. Despite decades of research, misunderstandings and erroneous beliefs surrounding the value of palliative care, particularly in the inpatient setting, have been pervasive. Grunauer and Mikesell [(33), p. 3] listed the more prevalent misconceptions regarding the implementation of palliative care in intensive care units as:

[It] is (1) ineffective and unimportant for most ICU patients, (2) synonymous with hospice and hopeless on the part of the family, patients, and/or clinician, (3) equivalent to the “soft skills” that health-care professionals already innately have, and (4) wasteful in that it absorbs valuable time and resources from intensivists.

However, they went on to state that none of these misconceptions were supported by research. In fact, multiple studies show that initiation of palliative care in a timely manner can improve symptom control, professional communication, the patient's perception of their quality of life, and even lengthen survival times (33). Dispelling the myths about palliative care and holistic care for neonates and infants with life limiting conditions and their families can be accomplished with ongoing education provided to the entire caregiving staff within each unit (18, 33, 34). More research and widespread education can enhance the appreciation of palliative care, which can foster a benevolent environment. Dr. Elvira Parravicini, M.D., neonatologist from Columbia University Irving Medical Center, said that “most obstetricians have come along to supporting us after seeing the benefits and appreciation from the patient of what we have done for them.”

### Professional Strength

Professional caregivers experiencing distress and high levels of stress have been associated with the development of cynicism, depersonalization of patients, diminished professional satisfaction, impaired decision making, medical errors, increased rates of adverse events, and higher mental and physical morbidities (9, 10, 35). Compassion fatigue or secondary traumatic stress disorder can also occur when empathetic caregivers experience, through a phenomenon known as countertransference, the trauma of their patients. Figley (36) coined the term *compassion fatigue* when he examined his own reaction to caring for military veterans debriefing from their traumatic combat experiences. He (36) said this regarding the personal anguish he encountered: “We feel the feelings of our clients. We experience their fears. We dream their dreams. Eventually, we lose a certain spark of optimism, humor and hope. We tire. We aren't sick, but we are not ourselves.”

Palliative and hospice professionals have higher rates of burnout and compassion fatigue than most other specialists with rates averaging as much as 61% in some studies (9, 37). Increased levels of distress and burnout can lead to absenteeism and negatively impact the palliative care team (38). Efforts to mitigate burnout and compassion fatigue are not without significant benefits for both the patient and the caregiver (39). Long-term job satisfaction can also flourish from relational efficacy (40).

### Fostering Resilience

Hernandez et al. (41) revealed that vicarious resilience occurs when professional caregivers experience their own growth through the provision of care and witness of patients enduring and recovering from traumatic experiences. Author van Dernoot Lipsky [(42), p. 11] wrote, “Those who support trauma stewardship believe that joy and pain are realities of life, and that suffering can be transformed into meaningful growth and healing when a quality of presence is cultivated and maintained even in the face of great suffering.” Therefore, a self-assessment to determine one's level of compassion fatigue or professional satisfaction is essential. The Professional Quality of Life Measure (ProQol) is a tool found to be useful for this appraisal (43). Self-care has been found to be a key aspect in developing resilience and maintaining an instrumental presence in the midst of suffering and loss (10, 35, 44, 45).

Resilience is an individual's ability to cope with adversity and maintain a healthy response to stressors (46). Effective coping and resilience can be nurtured by participation in a variety of self-care practices such as physical activity, mindful focusing, meditation, breathing exercises, and positive interactions with colleagues and friends (47). Hutti [(48), p. 25] found that “nurses reported strength to cope primarily from three sources: faith, relationships with fellow health care providers, and their own families.” Active involvement in a range of personal and employer organized supportive care strategies can augment coping, effective decision-making, and ultimately caregiver resilience in the midst of stressful workplace conditions (40). Dr. Parravinici said in regards to her comfort care team professionals, “We are friends, a tight knit group, and are not afraid to express concerns to each other. My friends care for my psychological needs. For instance, my nurse coordinator tells me things like ‘slow down,' ‘go home,' or ‘be realistic' when I need to hear it.”

A cohesive team is essential to the delivery of effective and beneficial palliative care in the perinatal/neonatal setting. Teams that have a well-defined program mission, vision, and goals that are fully embraced by each of the constituents are in an advantageous position to provide the necessary support for both individual and team resilience (11, 49). When the role of each team member is clearly delineated and in agreement with shared aspects of palliative care, the team as a whole benefits (11). Other aspects that can define the efficacy of a team include clear workload or productivity expectations, established lines of accountability, and a constructive evaluation process (49). Fostering respect and appreciation of the individual team members and the team as a whole requires evolving trust, open communication, prompt conflict resolution, and a shared movement toward common goals. Tempering factors that impair team function such as absenteeism, ongoing conflict, poor team structure, insufficient training, fear of conflict, and lack of commitment and accountability can help mitigate team erosion (49).

Studies have shown that caseloads perceived to be manageable by care providers often coincide with professional satisfaction and resilience. Keeton and colleagues [(50), p. 954] said that the “strongest single predictor of emotional resilience and personal accomplishment was control over schedule and hours worked.” Burnout and emotional exhaustion have been attributed to an increased workload (31, 51, 52). Specifically, palliative care providers reported more difficulty managing increasing caseloads because of the challenges in estimating the length of time needed for the provision of individual care plans, locating the family members involved in the care, and collaborating with the other team members (47, 53). In order to address issues related to absenteeism, employee retention, and burnout, healthcare institutions and legislative entities should carefully examine staffing polices and reimbursement guidelines (9).

### Educational Strategies

Lack of education and training has frequently been cited as a barrier to the provision of consistent and comprehensive palliative and bereavement support in the neonatal setting (7, 54–57). Haug et al. (55) demonstrated, in a nationwide study, that fewer than a third of institutions offer any formal training for physicians or staff on perinatal palliative or comfort care. However, research has shown that professionals exhibit better emotional coping when they have been provided with education related to bereavement and end-of-life issues (56, 58–60). For example, a strong majority (91.8%) of neonatologists involved in that study indicated that increased education in perinatal palliative and end-of-life care would be beneficial (55). Elements of self-care activity, as well as the symptoms and risk factors for both burnout and compassion fatigue, should be included in the education for perinatal palliative care professionals (7, 61). An enhanced knowledge base can increase caregiver confidence when serving patients.

A commitment to continuous learning is key to providing the best possible care for patients (62). “Staff education should be provided during orientation and then periodically throughout the course of every year” [(7), p. S30]. Palliative and bereavement care training with a multifaceted approach improves caregiver communication and confidence. Allyson McCullough, Registered Nurse and Bereavement Care Coordinator at Bella Natural Women's Care & Family Medicine and former Bereavement Care Coordinator on an inpatient maternity ward, said, “As a new nurse, I was uncomfortable … and felt that I needed to fill the silence with saying the right thing.” She went on to explain that education and experience have improved her confidence in communicating with and comforting patients.

Wilkinson and Roberts (63) demonstrated that it is most beneficial to use a range of teaching methods to nurture effective communication techniques. Utilizing experts for on-site training to facilitate group analyses of case studies, role-playing exercises, and demonstrations is an effective means of teaching professional caregivers techniques that will improve their comfort level in delivering bad news and confidence in supporting the family. According to Sweeney et al. [(64), p. 14], role-play is particularly impactful, stating:

[It] led to increased understanding of and changes in attitudes toward key palliative care principles, interdisciplinary teamwork, and communication of bad news. There was evidence of increased self-awareness. Findings suggest that the interdisciplinary breaking bad news role-play was a [particularly] rich integrative learning experience valued by students.

Professional caregivers who communicate effectively with parents facing a life-threatening illness in a neonate have been shown to have more professional confidence and better patient rapport. Increasing self-awareness, practicing specific responses via role-play activities, improving eye contact and body language, and learning to mirror a patient's own language are key components of providing quality patient care. Boles [(65), p. 307] asserts that empathy, sensitivity, active listening, a calming presence, and advocacy are essential skills “all nurses and psychosocial staff are called to provide at various points in the patient and family's illness journey.” McCullough also said, “[it] is often not what you say, but [in] the things not said—the non-verbal communication [and] body language, sitting with the family, holding their hand, crying with them, sitting in silence, and meeting them where they are.” Caregivers can provide an environment conducive to emotional healing by acknowledging the individual's feelings, withholding judgmental comments, reassuring them that they are not alone, and essentially giving them permission to grieve.

Patients and families grappling with the life-limiting diagnosis of an unborn or newly born child often report feeling powerless and marginalized in their involvement of care for their child (18). The provision of practical applications to assist the family bonding with and memorializing their severely ill or deceased neonate can empower both the bereaved and the caregivers. Providing personalized care that is directed in part by the patient or parent also fosters relational efficacy and provider resilience (40). Côté-Arsenault (2) said, “Parents in [her] study chose to focus their love and attention on what they could do at the present moment and on memory making,” which included memorialization, photography, and comfort measures. Examples of patient-focused care include recognizing physical cues from the baby and asking the parent to assist in determining their child's needs such as holding, skin-to-skin time, breastfeeding, and assessing for comfort, hunger, thirst, and cold (19).

### Formal and Informal Debriefing

Research indicates a beneficial advantage to members of a palliative team when they all participate as a group in scheduled discussions about the plan of care, appropriateness of the interventions, and the need for ongoing intensive support (39, 66, 67). Opportunities for formal debriefing should occur after every death and can be arranged to allow for reflection, quality improvement, shared narrative from the staff, and improved interdisciplinary cohesion (67). Many care teams have found that the utilization of a facilitator at these meetings can enhance the debriefing experience. For employers, it is important to note that stress management modalities and pre-incident education improve effective coping after encountering traumatic events (68).

Informal debriefing to a trusted colleague or mentor immediately or soon after a death or traumatic experience can also assist in mitigating the impact and facilitate personal emotional recovery (50, 69, 70). Also, small groups following a standardized debriefing process can help to ensure that all aspects of the incident were appropriately handled and allow for review from multiple perspectives (68). A nurse educator for the maternal ward at Flagstaff Medical Center reported, “I find that debriefing immediately after a case before going home helps me to relieve that pressure in my heart and in my mind.” She also reported that her employer had provided ample formal debriefing and mental health assistance, which she thought was indeed helpful for those caregivers needing more intense or prolonged assistance.

### Care Delivery Models

An institution should consider the manner in which perinatal palliative care is integrated with each unit. Currently there are two palliative care models integrated into the healthcare system. One model comprises a core group of professionals who provide consultation and care for families meeting the criteria for palliative care. Another model of care educates and trains the entire staff to provide palliative care (71). Casarett et al. [(71), p. 653] found that “families reported better outcomes when the patient received care in a palliative unit than they did when the patient received a palliative care consultation.” A pattern of favorable outcomes can fuel compassion satisfaction and foster resilience in the care provider.

In 2002, Catlin and Carter (28) deciphered that those staff members willing to provide perinatal palliative care should be the ones primarily involved in its provision rather than it being required of all in a given unit. However, Mancini (67) countered that all staff members should receive the training to care for any patient they are presented with including those needing end-of-life care. The physicians and nurses I interviewed reported that they had colleagues who had expressed their discomfort with the provision of palliative and end-of-life support and avoided assignments requiring it. A neonatal nurse from Flagstaff, Arizona, said in an interview, “having dedicated members is a challenge because that's usually not the reason why nurses went into this specialty [maternal/child nursing] in the first place … [and] it takes a toll [on the nurse] emotionally, physically, and psychologically.” Her statements underscore the need for increased training in palliative and end-of-life care early on in each professional education track. Ongoing education provided to the entire staff about palliative and end-of-life care should train and equip all staff with basic communication modalities to be compassionate and comforting in their interactions with patients. In 2019, Wool and Catlin [(34), p. S28] reported that “an integrated system of care increases quality and safety and contributes to patient satisfaction. The goal for respectful caregiving throughout the entire hospital system is achievable.” Professionals should feel free to disclose moral distress and receive the debriefing, spiritual, and emotional support necessary for a manageable resolution and promotion of ongoing occupational resilience (67).

In 2018, U.S. reproductive grief care experts Michaelene Fredenburg and Katie Geppert provided training for all staff members of the Ukrainian L'Viv Children's Hospital Perinatal Unit. The nurses who organized the event indicated that a high incidence of neonatal death and the staff's lack of knowledge about reproductive grief were the galvanizing motivations. The entire unit participated in the training including physicians, nurses, medical assistants, and anesthesiologists as well as social workers and a hospital chaplain. Nurses attending to the NICU patients also listened to the presentation on headsets.

Participants were introduced to research and concepts related to the unique nature of reproductive loss (5), disenfranchised grief (6), and the tasks of grieving (72). Evidence-based grief care best practices were shared as well as basic self-care ideas. Frequent staff debriefings were also recommended. After an uncomfortable amount of silence during the time allotted for questions, one of the nurses ventured to admit, “Please don't interpret our silence as disagreement, it's just that we culturally haven't been allowed to discuss this topic. Thank you for giving us the words and for giving us permission to talk to each other and to the parents of our patients.” Concertedly affirmed by the rest of the staff with applause and inquiries, the medical director pledged to adopt the recommended reproductive grief care and self-care policies. Months later, the nurses who had organized the in-service confirmed enhanced patient care and improved staff morale after the training.

### Self-Care Initiatives

Self-care is an imperative component to abating compassion fatigue (47, 73). One study reported that only 11% of the participating nurses and doctors practiced self-care [(74), p. 628]. Only 6% of that study's participants had an active self-care plan, and of those respondents “100% reported that they found the use of a self-care plan to be an effective self-care strategy” [(74), p. 627]. Koch and Jones [(30), p. 10] reported, “self-care is not only preventative for practitioners but can serve as a model for family caregivers about the critical importance of self-compassion and care.” Earnest intention to care for oneself can, in turn, result in an enhanced capacity for empathy and the service of others. Progressive resilience can be cultivated through a concerted and intentional effort to develop, implement, and fulfill a self-care plan.

The care provider should make an inventory of aspects of their personal and work practices that contribute to both distress and wellness (40, 46). The dimensions of holistic-wellness plans are multifactorial requiring attention to the mind, body, spirituality, and emotions (47, 75–77). Initially, a plan that includes pertinent goals and methods of accountability should be strategically developed. The anticipation of potential barriers can help the individual avoid being derailed from their pursuit (78). Diligent attention to potential subversions can foster timely attainment of the desired outcomes (79). Enlisting the assistance of a supportive person(s) to partner in the interventions and process of evaluating goals can improve accountability and overall beneficence (80). Care providers find that the creation of a written plan or contract for themselves is a motivating factor for their intended endeavor.

A cadre of wellness programs are often available at larger institutions. Kase et al. [(76), p. 3] found that only 60% of pediatric physicians had participated in those programs and their reasons for not partaking in them included “inconvenience of scheduling” (45.3%), time constraints (27.2%), the preference of participants to “handle things on my own” (34.1%), and the sentiment that partaking in the activity would not be helpful (21.9%). Studies have found that “one-size-fits-all” wellness programs offered by an institution might not effectively address specific needs of their employees and professional affiliates (76, 78, 79). Parravicini concurred, stating, “What people find to be helpful for self-care is a highly individual matter. Some prefer tennis, some skiing, some a walk in the park, others getting a beer.” Individualized discussions regarding the implementation of self-care strategies that include goals to be accomplished outside the work environment and continuing education can be integrated into occupational debriefing and evaluation conversations. Use of a self-administered assessment tool such as the Professional Quality of Life Assessment (43) or the Self-Care Assessment Worksheet (80) in planned intervals can assist in evaluating favorable progression toward the desired benefits (81).

A self-care plan would be ill equipped if it did not involve caring for personal losses. Caregivers cannot adequately assist others when they ignore their own bereavement, and they should not impose nor inject their personal grief experience into the care of others (82). James and Gilliand (82) observed that the palliative care provider can experience bereavement challenges when they encounter multiple meaningful losses in their domestic and occupational environments. They can find themselves overloaded emotionally and need more time to invest in adequate bereavement (82, 83). Obstacles to adequate grieving can occur when the nature of the loss is ambiguous. The absence of physical reminders, shared memories with others, and memorialization of the loss contribute to ambiguity and carrying out of the tasks associated with a typical grieving trajectory (5, 6, 72). When the grieving individual assimilates a healthier pattern of mourning losses through bereavement education and assistance “the knowledge and perspective gained from one's own growth following grief should serve as a quiet reservoir of strength” [(82), p. 461].

### Cultural and Spiritual Humility

A humble approach to assess the particular relational, emotional, cultural, and spiritual needs of the patient and family receiving palliative care is essential. In the midst of the suffering inherent to neonatal end-of-life care, healthcare professionals can have a human but irrational tendency to deduce that their clients' anguish could have been avoided with more expert care (11). A reverent but curious approach with not only patients and their families but also with other consulting or attending providers can enhance open, nonjudgmental communication and collaboration (84). Physician and inspirational author Coulehan [(85), p. 206] wrote:

[An] operational definition of medical humility includes four distinct but closely related personal characteristics that are central to good doctoring: unpretentious openness, honest self-disclosure, avoidance of arrogance, and modulation of self-interest. Humility, like other virtues, is best taught by means of narrative and role modeling. We may rightly be proud of contemporary medical advances, while at the same time experiencing gratitude and humility as healers.

In addition, Sasagawa and Amieux [(86), p. 925], researchers of multidisciplinary care collaboration, found that “both quantitative and qualitative analyses revealed that humility was viewed as an important character trait for successful inter-professional collaboration.” Dr. Parravicini exemplified an unassuming approach to patient assessment when she said, “I have to look at each single patient, look to them to tell me through objective signs and cues what to do for them. It is a tremendous gift to learn from my little patients and from their parents.”

Culturally sensitive care is necessary for families enduring the loss of an infant. Caregivers need to be alert and respectful to their clients' cultural, racial, ethnic, spiritual, linguistic, educational, and geographical differences. Cultural competency is a process of gathering knowledge about particular cultural groups; cultural humility is a process of inquiry and interpersonal curiosity approached with openness toward another (87). The pervasive nature of multiculturalism in the United States has made basing cultural competency on knowledge alone increasingly challenging (88). Yancu and Farmer [(89), p. e1–e2] said:

In practice, cultural diversity is manifested in a broad range of ever-changing behaviors, beliefs, rituals, restrictions, traditions, norms, institutions, and relationships that form the basis of cultural knowledge. This makes cultural mastery something akin to trying to grab onto a cloud.

In response to the change, cultural humility is quickly becoming the preferred style to assessing and addressing the cultural needs of patients (90).

Cultural and spiritual humility are conducive to superlative interpersonal communication and relational efficacy contributing to provider compassion satisfaction (88). Nurse researcher Kalu (91) concluded that religious and spiritual beliefs can support and promote better coping for individuals experiencing a reproductive loss. In her study done in Africa, one woman said, “The midwife sat and listened to me. She was very knowledgeable, spiritual and caring. She asked me if I wanted to see the baby. I said yes and spent time with [her]. I have a good memory of [my baby]. That helped me to find ways to adjust and move on knowing that the baby will always be part of my family” [(91), p. 6]. The inference from this patient exemplifies how a provider's reserved but open communication style can support a patient's spiritual beliefs without imposing any religious maxims.

Awareness of one's personal cultural and spiritual values enhances the caregiver's ability to accommodate multicultural and multiracial differences in the patients they serve (90, 92). Appreciation and practice of one's spirituality can promote professional resilience (40, 47, 93, 94). Belcher and Griffiths [(95), p. 271] said that:

Personal spirituality and a knowledge base to support spiritual caregiving were significant factors in hospice nursing staff members' competence and confidence in providing this intimate level of care. Respondents related a commitment to, and the ability to achieve, a level of spiritual care that was highly consistent with the spiritual needs of patients: the need for meaning and purpose in life, the need to give love, the need to receive love, and the need for hope and creativity.

Exploration of one's own cultural and spiritual background, particularly in aspects of family, childbearing, and grieving, can be a journey that enhances receptivity and caregiving satisfaction.

### Research and Practice Implications

Larger scale qualitative studies would make a valuable contribution to explicit needs of perinatal palliative care providers particularly in the aspects of occupational misconceptions, moral distress, aspects of humility, and professional resiliency. Quantitative studies on the effectiveness of more widespread bereavement education on patient and professional satisfaction indicators throughout a hospital system could potentially help healthcare systems to mitigate caregiver burnout and turnover. Qualitative and quantitative studies utilized to explore the efficacy of team enhancing interventions and structured debriefing processes may also yield benefits for institutions.

Perinatal palliative care is greatly valued in the lives of those grappling with life-limiting neonatal conditions. Support for those gifted and compassionate professionals who provide the caregiving needs to be strategic and multifaceted. The onus to implement salient measures to cultivate resilience in the perinatal palliative caregiver should not be only upon the individuals themselves, but also upon prevailing regulatory governing bodies and healthcare institutions as well. The implementation of efficacious staffing, debriefing protocols, and ongoing bereavement and self-care education are also vital components to resiliency.

There is a societal trend to minimize the impact of death for those that occur within the perinatal period. It is often erroneously believed that suffering and grieving is obsolete because the unborn or newly born child has not lived long enough to establish themselves. The assumption and commentary that one can “just move on and try to have another one” is incredibly hurtful to those mourning the loss of their child. Perinatal palliative care providers support and become that benevolent community necessary for families to endure and mourn. Parravicini said (96) in an interview, “I'm most proud of the babies because they are so abandoned to our care. In fact, what is unique in our program is that we always put ourselves in the babies' shoes.” She incites a professional humility that can assist perinatal palliative care providers to flourish within the setting of illness and death. “In its relational aspect, humility includes reverence or awe for the grace and strength of patients and their care-givers, a sense that the care-provider is not self-sufficient but needs the care-receiver” [(97), p. 291].

Byock [(98), p. 10] was posed the question from a woman with a terminal diagnosis, “Do I need to die well?” He went on to clarify that by promoting palliative care he had “hoped to dispel the notion that life ends the moment you receive a lousy diagnosis” [(98), p. 11]. He also profoundly concluded that, “it is possible to feel well within oneself and right with the world, even as one dies” and “that therein lies the hope for us all.” [(98), p. 13] The profession of caregiving has traditionally been a trajectory of hope for the future that involves a relationship between the patient and provider. However, in palliative care the aim of care provision is not focused on curing the ailment but on bettering the course of the illness. Professional and personal strengths rise to accommodate the vulnerabilities inherent to perinatal palliative care, but that relational efficacy requires concerted fortification.

## Author's Note

Professionals working in perinatal palliative care are at risk for burnout and compassion fatigue because of the emotional, collaborative, and ethical demands associated with the birth and death of infants with life-limiting illnesses. Consequently, healthcare institutions with perinatal palliative programs may be more vulnerable to poor retention, absenteeism, and waning quality of care. Medical professionals often report a lack of knowledge, inexperience in using effective communication skills (i.e., fear of saying the wrong thing), and emotional anguish concurrent with compassion fatigue as reasons for their emotionally avoidant or even irreverent behaviors in bereavement care delivery. Existential suffering and moral distress are common challenges inherent to the provision of care for life limiting neonatal conditions. There is an increased need for physician and interdisciplinary healthcare provider education, training, and ongoing support that promotes beneficial palliative care in the perinatal setting. Support should be to be strategic and multifaceted. The implementation of bereavement care training, effective communication modalities, and evidenced based practical applications are critical components for a thriving perinatal palliative care team. Authentic formal and informal debriefing, peer mentoring, adequate caseloads, robust provider self-care practices, cultural and spiritual humility can foster personal growth and vicarious resilience for perinatal palliative care professionals.

## Author Contributions

All authors listed have made a substantial, direct and intellectual contribution to the work, and approved it for publication.

## Conflict of Interest

KG was employed by the company Life Perspectives. MF was president and CEO of the company Life Perspectives. PJ was employed by the company Life Perspectives and the University of Akron. KJ was employed by the company Life Perspectives.
